# Extracellular Acidosis Promotes Metastatic Potency via Decrease of the *BMAL1* Circadian Clock Gene in Breast Cancer

**DOI:** 10.3390/cells9040989

**Published:** 2020-04-16

**Authors:** Yong-Jin Kwon, Eun-Bi Seo, Sun-Ho Kwon, Song-Hee Lee, Seul-Ki Kim, Sang Ki Park, Kyungjin Kim, SaeGwang Park, In-Chul Park, Jong-Wan Park, Sang-Kyu Ye

**Affiliations:** 1 Department of Pharmacology and Biomedical Sciences, Seoul National University College of Medicine, Seoul 03080, Korea; pistolhunter@snu.ac.kr (Y.-J.K.); lime872@snu.ac.kr (E.-B.S.); sunho180@snu.ac.kr (S.-H.K.); 24happy92@snu.ac.kr (S.-H.L.); cielryoma@snu.ac.kr (S.-K.K.); parkjw@snu.ac.kr (J.-W.P.); 2 Biomedical Science Project (BK21PLUS), Seoul National University College of Medicine, Seoul 03080, Korea; 3 Department of Life Sciences, Pohang University of Science and Technology, Pohang 37673, Korea; skpark@postech.ac.kr; 4 Department of Brain and Cognitive Sciences, Daegu Gyeongbuk Institute of Science and Technology, Daegu 42988, Korea; kyungjin@dgist.ac.kr; 5 Department of Microbiology and Immunology, INJE University College of Medicine, 633-165 GaegumDong, Busanjin Gu, Busan 614-735, Korea; micpsg@inje.ac.kr; 6 Division of Radiation Cancer Research, Korea Institute of Radiological and Medical Sciences, Nowon-gu, Seoul 01812, Korea; parkic@kirams.re.kr; 7 Ischemic/Hypoxic Disease Institute, Seoul National University College of Medicine, Seoul 03080, Korea; 8 Neuro-Immune Information Storage Network Research Center, Seoul National University College of Medicine, Seoul 03080, Korea

**Keywords:** circadian clock, BMAL1, hypoxia, acidosis, breast cancer, metastasis

## Abstract

Circadian oscillation is an essential process that influences many physiological and biological mechanisms and a decrease of circadian genes is associated with many diseases such as cancer. Despite many efforts to identify the detailed mechanism for decreasing circadian genes and recovering reduced circadian genes in cancer, it is still largely unknown. We found that BMAL1 was reduced in tumor hypoxia-induced acidosis, and recovered by selectively targeting acidic pH in breast cancer cell lines. Surprisingly, BMAL1 was reduced by decrease of protein stability as well as inhibition of transcription under acidosis. In addition, melatonin significantly prevented acidosis-mediated decrease of BMAL1 by inhibiting lactate dehydrogenase-A during hypoxia. Remarkably, acidosis-mediated metastasis was significantly alleviated by BMAL1 overexpression in breast cancer cells. We therefore suggest that tumor hypoxia-induced acidosis promotes metastatic potency by decreasing BMAL1, and that tumor acidosis could be a target for preventing breast cancer metastasis by sustaining BMAL1.

## 1. Introduction

Breast cancer is the most common cancer in females worldwide, and the second most cancer-related death reported annually [1,2]. Cancer metastasis represents progression of the primary cancer [3,4], with over 80% of breast cancer-related deaths diagnosed with metastasis [5]. Despite extensive studies of breast cancer, the incidence and metastasis rates have not been reduced [6]. A better understanding of cancer and new treatment strategies are therefore needed to prevent metastasis of breast cancer.

The circadian clock maintains daily oscillation rhythms with a 24 h periodicity in all living organisms. It responds to stimuli from external environments such as light, which affect pathological and physiological functions [7]. Several genes are associated with the circadian clock, including brain and muscle Arnt-like protein-1 (*BMAL1*), circadian locomotor output cycles kaput (*CLOCK*), period (*PER*s; Per1, Per2, and Per3), and cryptochromes (*CRY*s; Cry1 and Cry2), which form a complex network of transcription–translation feedback loops, post-translational modifications, and degradation [8,9].

Many people disrupt their daily circadian rhythm due to irregular patterns of life. Disrupted circadian rhythms are associated with a number of diseases, including cancer [10,11]. The cancer tissue has been shown to have lower expression of circadian genes than surrounding normal tissues, and more advanced states of cancer exhibit lower expression of circadian genes [12,13]. In addition, pattern of circadian genes expression was disrupted in MCF-7, MDA-MB-231, T47D, and Hs578T (breast cancer cell line) compared to MCF-10A (normal breast cell line) and HME1 (mammary epithelial cell line) [14,15]. BMAL1, which is one of the most important circadian clock genes, regulates overall circadian oscillations in humans, and previous reports have suggested that reduced BMAL1 is closely associated with tumor progression in cancer cells [16,17,18]. In particular, knocked-out BMAL1 significantly promoted tumor metastasis in MDA-MB-231 breast cancer cells [19]. Although BMAL1 is known to inhibit tumor progression, the mechanism for decreasing BMAL1 in cancer is largely unknown. Our aim was therefore to understand the relationship between breast cancer and circadian genes, and to identify tumor-mediated factors that reduce circadian genes.

In the previous study, it was reported that when melanoma cells were injected into mice skin, the rhythm patterns of clock genes were disrupted in the adjacent-tumor as well as the tumor, and it was suggested that the cause of this phenomenon is a change of the environment around the tumor [20]. Therefore, it can be expected that the tumor microenvironments will be significantly related to the disrupted circadian rhythms in the tumor. However, which tumor microenvironment caused the disruption of circadian rhythms is not well known yet. Therefore, we want to find out which tumor microenvironment reduces circadian genes and disrupts the circadian rhythms.

Hypoxia is a representative tumor microenvironment present in almost all solid tumors, including breast cancer, which forms a mass through abnormally rapid growth, and promotes cancer progression by regulation of angiogenesis, signaling molecules, and increased metabolism, and by changing the behavior of stromal cells surrounding the tumor [21,22,23]. Under conditions of sufficient oxygen, metabolic glycolysis generally relies on mitochondrial oxidative phosphorylation to generate ATP. However, during hypoxia, cancer cells increase in the presence of inefficient glycolysis because large amounts of ATP and building blocks are needed for cell proliferation. As a result, many byproducts such as lactic acid are produced and released from cells through plasma membrane transporters [24,25]. Finally, extracellular pH of cancer cells becomes acidic. Tumor acidosis, another major tumor microenvironment, promotes metastatic potency in MDA-MB-231 via the LAMP2 and ROS-AKT-NF-κB pathways [26,27,28]. Based on these studies, we speculated that tumor hypoxia and acidosis cause genetic alterations and it is closely related to the disruption of circadian rhythms in breast cancer.

Since the circadian rhythms were disrupted in most cancers, we hypothesized that common tumor microenvironments are the cause. In the previous study, the hypoxia-induced acidosis disrupts the circadian rhythms by mTOR signaling pathway [29]. However, in breast cancer, the relationship between the circadian gene BMAL1 and the tumor microenvironment is still largely unknown, and it is insufficient to explain only mTOR signaling pathway. In this study, we focused this phenomenon more on breast cancer and we aimed to determine the new pathway for decreasing BMAL1 and recovering reduced BMAL1 in the breast cancer microenvironment.

## 2. Materials and Methods

### 2.1. Reagents and Antibodies

Anti-BMAL1, anti-CLOCK, and anti-α-tubulin were purchased from Santa Cruz Biotechnology (Santa Cruz, CA, USA), anti-HIF-1α, anti-ZO-1, and anti-LDH-A were purchased from Cell Signaling Technology (Danvers, MA, USA). HRP-tagged anti-rabbit and anti-mouse were purchased from Enzo Life Science (Farmingdale, NY, USA). Lactic acid, cycloheximide (CHX), G418 disulfate salts (G418), sodium oxamate, melatonin, and sodium bicarbonate (NaHCO_3_) were purchased from Sigma Aldrich (St. Louis, MO, USA).

### 2.2. Cell Lines and Culture Conditions

The human normal breast cell line MCF-10A was purchased from the American Type Culture Collection (ATCC) and maintained in DMEM/F-12 (Welgene, Gyeongsan, Korea) supplemented with 5% horse serum (GIBCO, Waltham, MA, USA), 100 ng/mL cholera toxin, 20 ng/mL EGF, 0.5 mg/mL hydrocortisone, 10 μg/mL insulin, and 1% penicillin/streptomycin (Capricorn Scientific GmbH, Ebsdorfergrund, Germany). The human breast cancer cell lines MCF-7, T47D, ZR-75-1, MDA-MB-231, MDA-MB-468, and Hs578T were purchased from ATCC, and maintained in DMEM (Capricorn Scientific GmbH) supplemented with 10% fetal bovine serum (FBS, Capricorn Scientific GmbH) and 1% penicillin/streptomycin (Capricorn Scientific GmbH). The mouse mammary tumor cell line TUBO and TUBO-P2J were kindly provided from Professor SaeGwang Park (INJE University College of Medicine, Korea) and maintained in DMEM (Capricorn Scientific GmbH) supplemented with 10% FBS (Capricorn Scientific GmbH), 10% NCTC-109 medium (GIBCO), 2 mmol/L L-glutamine, 0.1 mmol/L MEM nonessential amino acids (GIBCO), and 1% penicillin/streptomycin (Capricorn Scientific GmbH). BMAL1 overexpressing MDA-MB-231 cell lines were established from G418-resistant clones. For hypoxia stimulation, the oxygen tension in incubator (Vision Science, Seoul, Korea) was 21% O_2_ normoxic condition and 2% O_2_ hypoxic condition respectively. These cells were maintained in humidified atmosphere containing 5% CO_2_ at 37 °C.

### 2.3. Conditioned Media

MCF7 and MDA-MB-231 cells were seeded onto the plate. When cells were attached, growth media was exchanged for serum free media, and then cultured under normoxia (21% O_2_) or hypoxia (2% O_2_) for 48 h, respectively. Normoxic conditioned media (NCM) were obtained after culturing cells under normoxia, and hypoxic conditioned media (HCM) were obtained after culturing cells under hypoxia. The heat-inactivated HCM were obtained by boiling HCM at 100 °C for 5 min to degrade all secretory proteins in cultured media, and the neutralized HCM were obtained by neutralizing acidified pH of HCM using 1 M NaOH dose-dependently. These conditioned media were treated in each cell line for 24 h, respectively.

### 2.4. pH Regulation

To acidify the media pH, 1 M HCl and lactic acid were treated in the media dose-dependent manner and incubated at 37 °C 5% CO_2_ condition for 24 h. After stabilization, pH of the media was immediately measured using a SevenEasy pH meter (Mettler Toledo, Columbus, OH, USA). The pH of the cultured media was measured immediately after the experiments using a SevenEasy pH meter, and the analyzed pH of the cultured media was summarized in Supplementary Tables S4–S6.

### 2.5. Cell Viability Assay

Cells were grown in 96-well culture plates with each condition, and incubated with MTT reagent for 4 h. Blue formazan crystals were solubilized with DMSO, and formazan levels were determined at 570 nm using an Infinite M200 PRO plate reader (Tecan Group Ltd., Männedorf, Switzerland).

### 2.6. DNA and siRNA Transfection

For overexpression of genes, cells were transfected with the following constructs: pEGFP-C3-vector and pEGFP-C3-BMAL1. pEGFP-C3-vector and pEGFP-C3-BMAL1 were kindly provided from Professor Sang Ki Park (Pohang University of Science and Technology, Korea). BMAL1, CLOCK, and LDH-A were knocked down by transfection with human si-BMAL1 (SI00023016 and SI00023037; Qiagen) mouse si-BMAL1 (SI02685865 and SI00166719; Qiagen), human si-CLOCK (SI00069769 and SI00069776; Qiagen), human si-LDH-A (siRNA no.3939-1; Bioneer), and a negative control si-RNA (1028290; Qiagen). Transfection was performed using the Lipofectamine 3000 and Lipofectamin RNAiMax reagent (Invitrogen), according to the manufacturer’s protocol.

### 2.7. Immunoblotting

Cells were washed cold PBS, and then lysed in the triton lysis buffer containing protease and phosphatase inhibitors. After incubation for 30 min on ice, lysates were centrifuged at 13,000 rpm for 20 min at 4 °C, and supernatants were collected. Lysates were separated on 7–12% SDS-polyacrylamide gels, and transferred to nitrocellulose membranes (GE Healthcare Life Sciences, Chicago, Illinois). Membranes were blocked in 5% skim-milk for 1 h and incubated with primary antibodies overnight at 4 °C. Membranes were incubated with a horseradish peroxidase-conjugated secondary antibodies for 1 h, and then visualized using the ECL detection kit (Young In Frontier Co., Ltd., Seoul, Korea). Densitometric measurements of Western blot bands were analyzed using the ImageJ program (Supplementary Figures S7–S11).

### 2.8. RNA Isolation and Quantitative Real-Time PCR

Total RNA was isolated using the RNAiso Plus reagent (Takara, Shiga, Japan) and cDNA was synthesized using a ReverTra Ace qPCR RT Master Mix (TOYOBO, Osaka, Japan). Quantitative real-time PCR was performed using the EvaGreen qPCR Mastermix (Applied Biological Materials, Richmond, BC, Canada), and fluorescence was detected by CFX Connect Real-Time PCR Detection System (Bio-Rad). Data were analyzed with the CFX Manager Software (Bio-Rad), and the mRNA values of targeted genes were normalized to tubulin expression. The sequences of PCR primers are summarized in Supplementary Table S3.

### 2.9. Trans-Well Migration Assay

Cells were cultured in cell culture inserts with an 8 μm pore size polycarbonate membrane (Corning Life Sciences, Tewksbury, MA, USA). Cells were seeded in the upper trans-well chamber containing serum-free media and the lower chamber contained complete media to induce cell migration. After incubation, cells on the upper chamber membrane were fixed and stained with Diff-Quick solution kit (Sysmex Corporation, Kobe, Japan). The stained cells on the upper side of the interface membrane were wiped with a cotton swab and migration cells on the lower side of the membrane were counted using ECLIPSE TS100 inverted microscope (Nikon Instruments Inc., Melville, NY, USA).

### 2.10. Wound-Healing Assay

Cells were seeded onto 6-well plate. When the cell confluence reached about 80–90%, detached cells were scratched using a 200 μL plastic tip, and then the debris was removed by washing with PBS. The cells were incubated in serum-free medium. The cell migration ability was analyzed by visualizing the edge of cells using ECLIPSE TS100 inverted microscope (Nikon Instruments Inc., Melville, NY, USA).

### 2.11. Public Datasets

Breast cancer datasets used in this study were GSE3744 and GSE5364. An online Kaplan–Meier (KM) plotter database was used to analyze recurrence free survival (RFS) of breast cancer patients (http://kmplot.com/analysis) [30].

### 2.12. Statistical Analysis

All data were statistically analyzed using Microsoft Excel 2017 software and Graph pad Prism 5 software. Results are presented as means ± standard deviation (SD) of three independent experiments. The statistical significance was determined by an unpaired Student’s *t*-test. All statistical significances were considered when a *p*-value was less than 0.05.

## 3. Results

### 3.1. Chronic Hypoxia Reduces the BMAL1 Expression in Breast Cancer Cells

**Figure 1:**
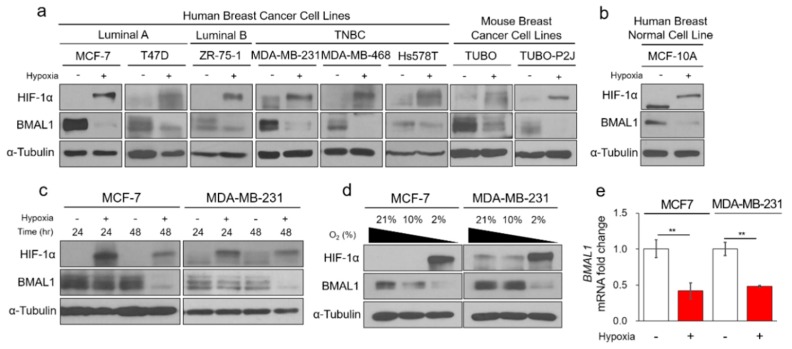
Chronic hypoxia reduces circadian BMAL1 expression in breast cancer cells. (**a**) Breast cancer cell lines were incubated in normoxia or 2% O_2_ hypoxia for 48 h. Cell lysates were analyzed by immunoblotting. (**b**) MCF-10A was incubated in normoxia or 2% O_2_ hypoxia for 48 h. Cell lysates were analyzed by immunoblotting. (**c**) MCF-7 and MDA-MB-231 were incubated in normoxia or 2% O_2_ hypoxia for 24 and 48 h. Cell lysates were analyzed by immunoblotting. (**d**) MCF-7 and MDA-MB-231 were incubated in normoxia, 2% or 10% O_2_ hypoxia for 48 h. Cell lysates were analyzed by immunoblotting. (**e**) MCF-7 and MDA-MB-231 were incubated in normoxia or 2% O_2_ hypoxia for 48 h. Cell lysates were analyzed by RT-qPCR. Data represent the mean ± SD, *n* = 3. ** *p* < 0.01 vs. the control group by a Student’s *t*-test.

Since previous studies have reported that circadian genes including BMAL1 are reduced in almost cancers, we hypothesized that tumor hypoxia, a common and predominant occurrence in cancer, plays a critical role in decrease of the BMAL1 circadian gene. Hypoxia significantly reduces BMAL1 protein expression in MCF-7, T47D (luminal A), ZR-75-1 (luminal B), MDA-MB-231, MDA-MB-468, Hs578T (basal-like) human breast cancer, and TUBO and TUBO-P2J (TUBO metastatic variant) mouse breast cancer cells (Figure 1a and Supplementary Table S1). In addition, BMAL1 was reduced under hypoxia in MCF-10A human normal breast cells (Figure 1b). Since MCF-7 and MDA-MB-231 cell lines have been used for decades as in vitro breast cancer models, we used for these representative two cell lines. BMAL1 is only reduced in chronic and deep hypoxia for 48 h in 2% O_2_, and not in acute and mild hypoxia for 24 h in 2% O_2_ or 48 h in 10% O_2_ (Figure 1c,d). In addition, hypoxia also reduces BMAL1 mRNA expression (Figure 1e). CLOCK is also the circadian gene and forms a complex with BMAL1. This heterodimer complex binds to E-box elements in promoters and drives circadian rhythms [8]. The circadian genes are linked to other circadian genes expression because these are composed of transcription–translation feedback loops [9]. Interestingly, protein and mRNA levels of CLOCK were also reduced under chronic hypoxia (Supplementary Figure S1a,b). In this study, we focused more on BMAL1 among several clock genes. Hypoxia-inducible factors (HIFs) are transcription factors that are activated in low oxygen conditions. The previous studies have reported that not only hypoxia but also HIF-1α reciprocally regulates circadian rhythms in human osteosarcoma cells and mouse myoblast cells [29,30,31]. However, in breast cancer cells, we found that the hypoxia-mimetic agent CoCl_2_ did not affect BMAL1 protein expression (Supplementary Figure S1c). In addition, both overexpression of wild-type (WT) and constitutively stable HIF-1α (P405A and P564A) did not affect the BMAL1 protein expression (Supplementary Figure S1d). Moreover, when HIF-1α was silenced during hypoxia, it did not prevent the decrease of BMAL1 protein expression (Supplementary Figure S1e). Together, these results suggested that BMAL1 was reduced by chronic hypoxia independently of HIF-1α in breast cancer cells.

### 3.2. Hypoxia-Mediated Acidosis Reduces Circadian BMAL1 Expression in Breast Cancer Cells

**Figure 2:**
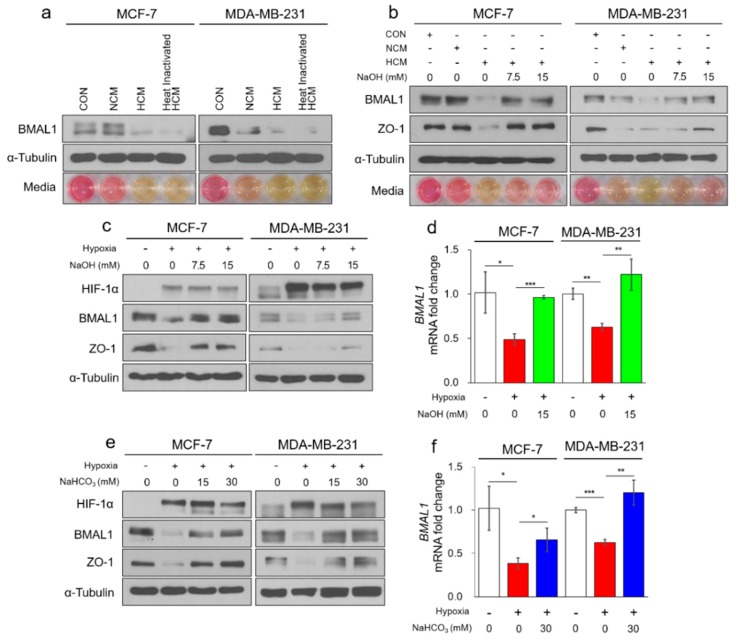
Hypoxia-mediated acidosis reduces circadian BMAL1 expression in breast cancer cells. (**a**) MCF-7 and MDA-MB-231 were treated with fresh media (control; CON), normoxic conditioned media (NCM), hypoxic conditioned media (HCM), or heat inactivated HCM for 24 h. Cell lysates were analyzed by immunoblotting. Representative images of cultured media are shown (bottom panel). (**b**) MCF-7 and MDA-MB-231 were treated with fresh media, NCM, HCM, or NaOH treated HCM for 24 h. Cell lysates were analyzed by immunoblotting. Representative images of cultured media are shown (bottom panel). (**c**,**d**) MCF-7 and MDA-MB-231 were incubated in normoxia or 2% O_2_ hypoxia with NaOH for 48 h. Cell lysates were analyzed by immunoblotting (**c**) and RT-qPCR (**d**). (**e**,**f**) MCF-7 and MDA-MB-231 were incubated in normoxia or 2% O_2_ hypoxia with NaHCO_3_ for 48 h. Cell lysates were analyzed by immunoblotting (**e**) and RT-qPCR (f). Data represent the mean ± SD, *n* = 3. * *p* < 0.05, ** *p* < 0.01 and *** *p* < 0.001 vs. the control group or between two groups by a Student’s *t*-test.

In hypoxia, many cells secrete protumorigenic cytokines, chemokines, and growth factors, which play important roles in tumorigenesis, such as recruiting diverse types of immune cells and accelerating angiogenesis [22,24]. We hypothesized that hypoxia-mediated secretory factors reduce BMAL1 expression in breast cancer cells. When MCF-7 and MDA-MB-231 breast cancer cells were exposed to hypoxic conditioned media (HCM), BMAL1 was reduced (Figure 2a). However, BMAL1 was also reduced in heat-inactivated HCM, in which all secretory proteins were degraded (Figure 2a). These results suggested that the hypoxia-mediated secretory proteins had no effect on the regulation of BMAL1, but other sources of HCM may be involved. We then observed that media containing phenol red was yellowish in HCM-treated MCF-7 cells, normoxic conditioned media (NCM), and HCM-treated MDA-MB-231 cells (Figure 2a bottom). This phenomenon exhibited a similar pattern as the decrease of BMAL1 expression. Since yellowish medium indicates that acidosis has occurred and in the previous study, the BMAL1 was disrupted under hypoxia-induced acidosis in osteosarcoma cells [29], we expected that hypoxia-induced acidosis also reduced BMAL1 expression in breast cancer cells. BMAL1 was reduced by HCM, but was not reduced when the pH of the medium was neutralized by NaOH in a dose-dependent manner (Figure 2b and Supplementary Figure S2a). Many studies have reported that tight junction proteins are reduced in acidic conditions [32,33]. Since the tight junction protein ZO-1 was significantly reduced under acidic conditions, it was an indication that the breast cancer cells were involved in acidosis. In addition, the protein and mRNA levels of BMAL1 in MCF-7 and MDA-MB-231 breast cancer cells were reduced in chronic hypoxia but unchanged when the pH of the medium was neutralized by NaOH (Figure 2c,d) or buffered by NaHCO_3_ (Figure 2e,f and Supplementary Figure S2b). Together, these results showed that hypoxia-induced acidosis reduced BMAL1 expression, which could be prevented by selectively targeting the acidic pH in breast cancer cells.

### 3.3. Tumor Acidosis Reduces BMAL1 via Inhibition of Transcription Activity and Protein Stability in Breast Cancer Cells

**Figure 3:**
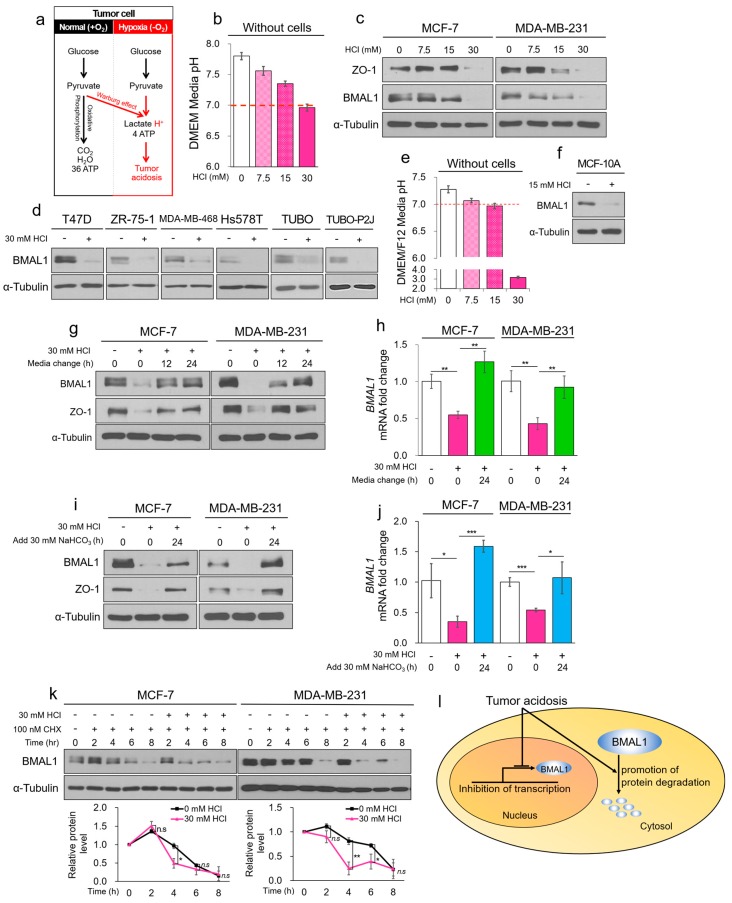
Tumor acidosis reduces BMAL1 via inhibition of transcriptional activity and protein stability in breast cancer cells. (**a**) The summarization of the glycolysis pathway in normoxia and hypoxia. (**b**) HCl-mediated acidic DMEM media were incubated for 24 h without cells, and media pH was immediately measured using a pH meter. (**c**,**d**) Breast cancer cell lines were treated with HCl-mediated acidic media for 24 h. Cell lysates were analyzed by immunoblotting. (**e**) HCl-mediated acidic DMEM/F12 media were incubated for 24 h without cells. pH of the cultured media was immediately measured using a pH meter. (**f**) MCF-10A was treated with HCl-mediated acidic media for 24 h. Cell lysate was analyzed by immunoblotting. (**g**,**h**) MCF-7 and MDA-MB-231 were treated with HCl-mediated acidic media for 24 h. The acidic cultured media were exchanged to fresh media and then incubated for 12 and 24 h. Cell lysates were analyzed by immunoblotting (**g**) and RT-qPCR (**h**). (**i**,**j**) MCF-7 and MDA-MB-231 were treated with HCl-mediated acidic media for 24 h. The acidic cultured media were added to NaHCO_3_ and then incubated for 24 h. Cell lysates were analyzed by immunoblotting (**i**) and RT-qPCR (**j**). (**k**) MCF-7 and MDA-MB-231 were treated with CHX in acidic condition for the indicated periods. Cell lysates were analyzed by immunoblotting. The blots of BMAL1 were quantified using ImageJ (bottom panel). (**l**) Graphical summarization of the dual pathways that reduce BMAL1. Data represent the mean ± SD, *n* = 3. * *p* < 0.05 and ** *p* < 0.01 vs. the control group or between two groups by a Student’s *t*-test.

In hypoxia, most cells including cancer cells release large amounts of lactic acid via anaerobic glycolysis and become acidified, which is a hallmark of tumor malignancy [26]. According to previous studies, in normal cells, intracellular pH (pHi) is lower than extracellular pH (pHe; pHi = 7.2 and pHe = 7.4). However, in cancer cells, pHi is higher than pHe (pHi ≥ 7.2 and pHe = 6.7–7.1) (Supplementary Table S2) [34,35,36,37,38,39]. We assumed the tumor acidic pHe to be < 7.0 according to several studies (Figure 3a).

To determine whether BMAL1 was reduced only by tumor acidosis, we adjusted the pH of the cell culture medium using HCl (Figure 3b). When the acidic media were treated in MCF-7 and MDA-MB-231 cells for 24 h, media with pH < 7.0 reduced BMAL1 expression, and the cultured media of MCF-7 and MDA-MB-231 cells had pH values less than 6.7 (Figure 3c and Supplementary Figure S3a,b). Similar results were also obtained for T47D, ZR-75-1, MDA-MB-468, Hs578T human breast cancer, and TUBO and TUBO-P2J mouse breast cancer cell lines (Figure 3d). We also found that BMAL1 was reduced by HCl-mediated acidosis in MCF-10A normal breast cells (Figure 3e,f and Supplementary Figure S3c). Since reduced circadian genes in all organs can directly cause many diseases including cancer, continuously oscillating circadian genes are important for the treatment and prevention of diseases [11]. Notably, a decrease of BMAL1 by tumor acidosis was recovered by the exchanging acidic cultured media to fresh media (Figure 3g,h) or adding NaHCO_3_ to the acidic cultured media (Figure 3i,j and Supplementary Figure S3d). Additionally, we found that CLOCK was also reduced by tumor acidosis (Supplementary Figure S3e,f). The metabolic byproduct lactic acid is a major metabolite that induces hypoxia-mediated acidosis. BMAL1 is also reduced by lactic acidosis and recovered by exchanging media (Supplementary Figure S3g–i) or adding NaHCO_3_ (Supplementary Figure S3j–k). These results suggested that circadian BMAL1 was only reduced by acidosis and recovered by neutralizing and buffering the acidic pH in breast cancer cell lines. Under hypoxia and acidic conditions, while the protein level of BMAL1 was almost reduced (Figure 1a and Figure 3c), the mRNA level was not completely reduced in breast cancer cells (Figure 1e and Figure 3h,j). Since previous studies have reported that circadian genes including BMAL1 sustaining their circadian rhythms through transcription, protein post-translational modification, and protein degradation [8], we hypothesized that other mechanisms may reduce BMAL1 protein levels by acidosis besides transcriptional inhibition. Cycloheximide (CHX), an inhibitor of de novo protein synthesis, decreased the half-life of BMAL1 protein expression in MCF-7 and MDA-MB-231 breast cancer cells (Supplementary Figure S3l). Notably, we found that the half-life of BMAL1 was shortened even further by acidic conditions and treatment with CHX (Figure 3k). Together, the results suggested that tumor acidosis reduced BMAL1 via inhibition of transcription and decrease of protein stability (Figure 3l).

### 3.4. Tumor Acidosis-Mediated Decrease of BMAL1 Promotes Metastatic Potency in Breast Cancer Cells

**Figure 4:**
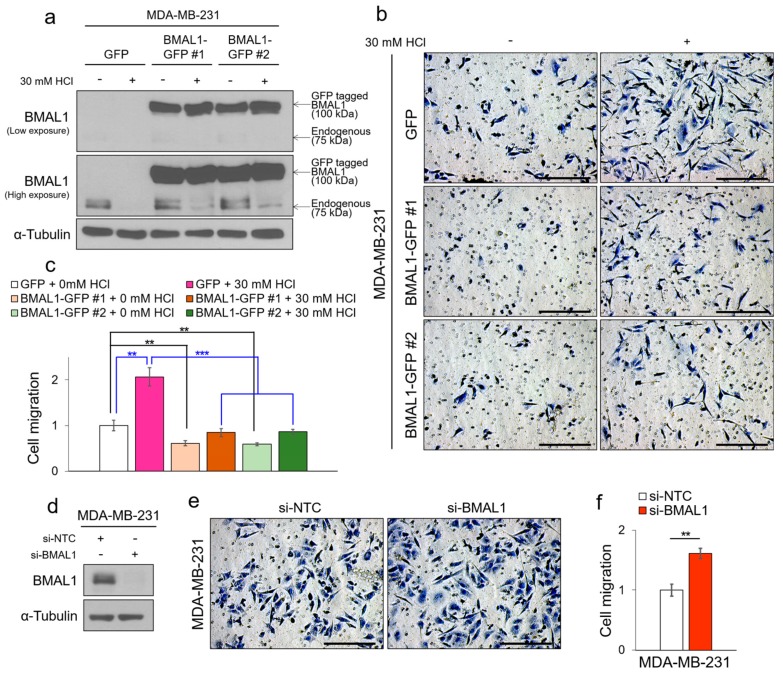
Tumor acidosis-mediated decrease of BMAL1 expression promotes metastatic potency in breast cancer cells. (**a**) The green fluorescent protein (GFP) or GFP-BMAL1 stably overexpressed MDA-MB-231 cell lines were incubated in HCl-mediated acidic condition for 24 h as indicated, and cell lysates were determined by immunoblotting. (**b**,**c**) GFP or GFP-BMAL1 stably overexpressed MDA-MB-231 cell lines were subjected to trans-well migration assay in HCl-mediated acidic condition for 24 h as indicated. Representative images of migrated cells are shown. Scale bars: 250 μm (**b**). The average number of migrated GFP or GFP-BMAL1 stably overexpressed MDA-MB-231 cell lines was counted in three random microscopic fields (**c**). (**d**) MDA-MB-231 was transfected with si-NTC or si-BMAL1. Cell lysates were determined by immunoblotting. (**e**,**f**) MDA-MB-231 was transfected with si-NTC or si-BMAL1, and was subjected to trans-well migration assay for 24 h. Representative images of migrated cells are shown. Scale bars: 250 μm (**e**). The average number of migrated MDA-MB-231 was counted in three random microscopic fields (**f**). Data represent the mean ± SD, *n* = 3. ** *p* < 0.01 and *** *p* < 0.001 vs. the control group or between two groups by a Student’s *t*-test.

Breast cancer can be successfully treated by surgery and therapeutic strategies, but when metastasis occurs, the survival rate falls dramatically despite many previous studies to prevent breast cancer metastasis [5]. A novel mechanism is therefore needed to prevent tumor metastasis. Since tumor hypoxia and acidosis are well-known to promote tumor metastasis in cancers including MDA-MB-231 cells, targeting the tumor hypoxia and acidosis is likely important in treating tumors [40,41,42,43]. Interestingly, the previous study reported that knocked-out BMAL1 promotes metastasis in MDA-MB-231 cells, and previously our results showed that BMAL1 was reduced by tumor hypoxia-induced acidosis and recovered by selectively targeting the acidic pH in breast cancer cells [19]. Based on these references and our results, we hypothesized that promoted metastasis under acidosis is caused by a decrease of BMAL1, and maintaining BMAL1 by buffering acidic pH is a therapeutic approach to prevent metastasis. We used MDA-MB-231 and TUBO-P2J breast cancer cell lines, which have metastatic potencies, to investigate the relationship between reduced BMAL1 by acidosis and breast cancer metastasis. MDA-MB-231 and TUBO-P2J breast cancer cells increase metastasis during acidosis. However, when the acidic pH was buffered, increased migration by acidosis was alleviated (Supplementary Figure S4a,b). This tendency was the same as the expression pattern of BMAL1 (Supplementary Figure S4c). To characterize the role of BMAL1 in acidosis-mediated breast cancer metastasis, we used an overexpression system to maintain BMAL1 expression levels during acidosis. Importantly, increased migration by acidosis was alleviated in the green fluorescent protein (GFP)-tagged BMAL1-transfected MDA-MB-231 and TUBO-P2J breast cancer cells because GFP-BMAL1 was overexpressed despite decrease of endogenous BMAL1 by acidosis (Supplementary Figure S4d–f). To confirm these results, we established GFP-BMAL1 stably overexpressed MDA-MB-231 cell lines. When GFP-tagged BMAL1 was consistently overexpressed, tumor migration was significantly alleviated, and increased migration by acidosis was also alleviated in GFP-BMAL1 stable MDA-MB-231 cell lines (Figure 4a–c, Supplementary Figure S4g). By contrast, when BMAL1 was knocked down using small interfering RNA, tumor migration was significantly promoted in MDA-MB-231 (Figure S4d–f) and TUBO-P2J breast cancer cell lines (Supplementary Figure S4h–j). Additionally, we found that knock-down of CLOCK promotes metastasis, and double knock-down of BMAL1 and CLOCK further promotes metastasis in MDA-MB-231 breast cancer cells (Supplementary Figure S4k–m). Together, these results suggest that tumor acidosis-mediated decrease of the BMAL1 promotes tumor metastatic potency in breast cancer cell lines, and selectively targeting tumor acidosis to maintain BMAL1 prevents breast cancer metastasis.

### 3.5. Melatonin Attenuates Decrease of BMAL1 by Inhibiting Hypoxia-Mediated LDH-A in Breast Cancer Cells

**Figure 5:**
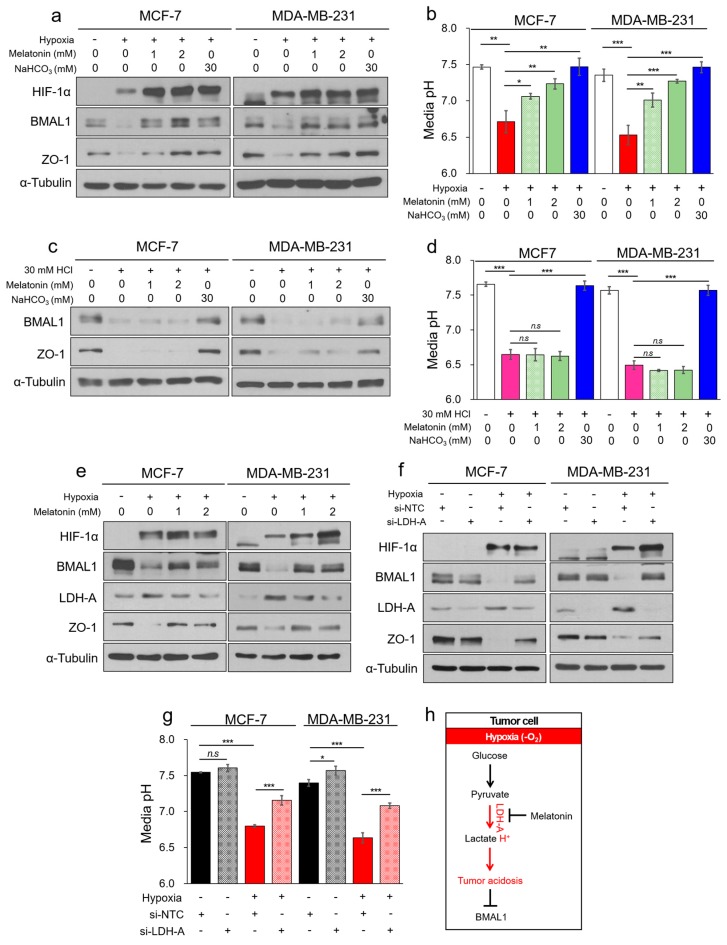
Melatonin attenuates decrease of BMAL1 expression by inhibiting hypoxia-mediated LDH-A in breast cancer cells. (**a**,**b**) MCF-7 and MDA-MB-231 were incubated in normoxia or 2% O_2_ hypoxia with melatonin or NaHCO_3_ for 48 h. Cell lysates were analyzed by immunoblotting (**a**) and pH of the cultured media was immediately measured using a pH meter (**b**). (**c**,**d**) MCF-7 and MDA-MB-231 were incubated in acidic condition with melatonin or NaHCO_3_ for 24 h. Cell lysates were analyzed by immunoblotting (**c**) and pH of the cultured media was immediately measured using a pH meter (**d**). (**e**) MCF-7 and MDA-MB-231 were incubated in normoxia or 2% O_2_ hypoxia with melatonin for 48 h. Cell lysates were analyzed by immunoblotting. (**f**,**g**) MCF-7 and MDA-MB-231 were transfected with si-NTC or si-LDH-A, and cells were incubated in normoxia or 2% O_2_ hypoxia for 48 h. Cell lysates were determined by immunoblotting (**f**) and pH of the cultured media was immediately measured using a pH meter (**g**). (**h**) The summarization of pathway that melatonin attenuates acidosis-mediated decrease of BMAL1 by inhibiting LDH-A in hypoxia. Data represent the mean ± SD, *n* = 3. * *p* < 0.05, ** *p* < 0.01, and *** *p* < 0.001 vs. the control group or between two groups by a Student’s *t*-test.

Since BMAL1 was reduced by tumor acidosis, we wanted to identify a biological mechanism to prevent the decrease of BMAL1 during tumor acidosis. Melatonin, a hormone produced in the pineal gland, is responsible for the oscillation of overall circadian rhythms in humans [44]. Melatonin regulates the sleep–wake cycle, as well as blood pressure and body temperature [45,46,47], and has been also reported to be a potential effector of antioxidant, anti-inflammatory, and anticancer activities in many diseases [48,49,50]. We hypothesized that melatonin might prevent the decrease of BMAL1 by tumor acidosis. To investigate a possible mechanism, MCF-7 and MDA-MB-231 cells were treated with melatonin in hypoxic conditions. Remarkably, with melatonin treatment, hypoxia-mediated decrease of BMAL1 was significantly prevented (Figure 5a,b and Supplementary Figure S5a,b). However, in HCl-induced acidic conditions, decrease of BMAL1 was not prevented by melatonin because the HCl-induced acidic pH was not controlled by melatonin in MCF-7 and MDA-MB-231 cells (Figure 5c,d and Supplementary Figure S5c) and without cells (Supplementary Figure S5d,e). These results suggest that melatonin prevented hypoxia-induced acidosis and a decrease of BMAL1 in breast cancer cells. During anaerobic glycolysis, pyruvate is converted into lactic acid by LDH, the primary enzyme of hypoxia-mediated acidosis [51]. Previous studies reported that melatonin inhibits LDH expression and activity [52,53,54]. Based on these results, we hypothesized that increased LDH in hypoxia was inhibited by melatonin, which might prevent hypoxia-mediated decrease of BMAL1 by inhibiting acidosis. As expected, increased LDH-A in hypoxia was reduced by melatonin in MCF-7 and MDA-MB-231 breast cancer cells (Figure 5e). Additionally, we confirmed that hypoxia-mediated decrease of BMAL1 was prevented by inhibiting LDH-A and tumor acidosis using oxamate (Supplementary Figure S5f,g), non-competitive LDH inhibitor, and small interfering RNA (Figure 5f,g). LDH-A is known as HIF-1α target gene for decades. However, in a previous study, overexpression of WT HIF-1α did not adequately increase the several HIF-1α target genes including LDH-A compared to hypoxia [55]. We additionally confirmed that HIF-1α alone could not regulate LDH-A and cultured media pH (Supplementary Figure S5h,i). Our results suggest that the hypoxia-induced acidosis reduced BMAL1 independently of HIF-1α in breast cancer cells. Together, these results show that melatonin maintained BMAL1 expression by inhibiting the expression of LDH-A to prevent hypoxia-induced acidosis in MCF-7 and MDA-MB-231 breast cancer cells (Figure 5h). Therefore, we proposed a new mechanism for melatonin, which regulates BMAL1 expression during hypoxia-mediated tumor acidosis by inhibiting LDH-A.

### 3.6. Decrease of BMAL1 is Clinically Related to Poor Prognoses in Breast Cancer Patients

**Figure 6:**
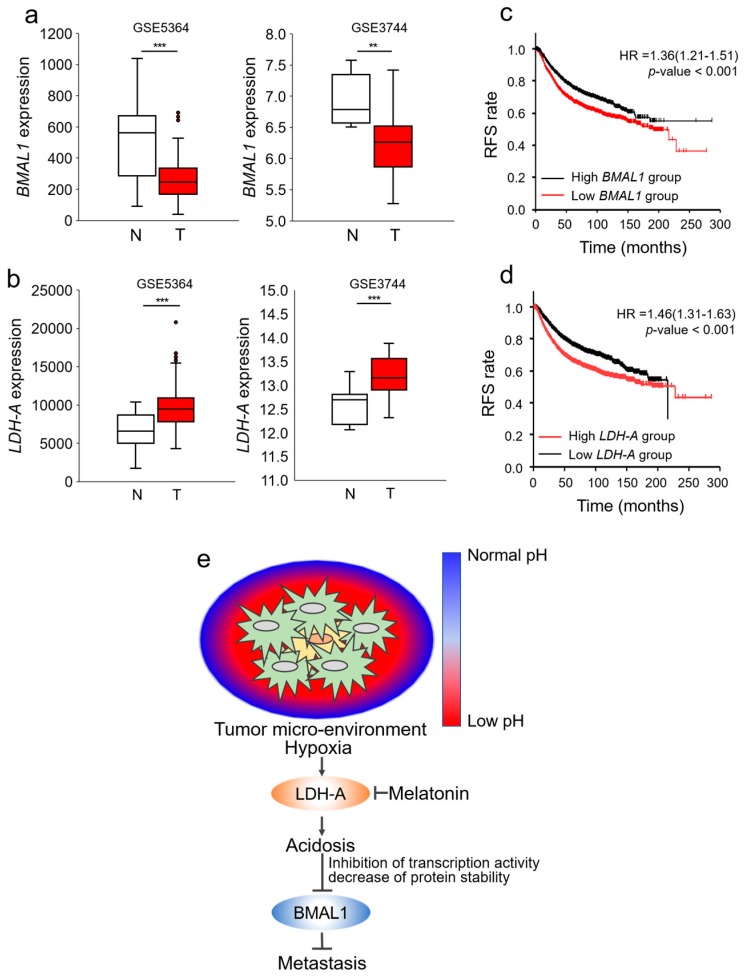
Decrease of BMAL1 is clinically related to poor prognoses in breast cancer patients. (**a**,**b**) BMAL1 (**a**) and LDH-A (**b**) mRNA expression in normal and cancer breast tissue samples from GSE536 and GSE3744 database sets. N: normal breast tissue T: breast cancer tissue. (**c**,**d**) Relapse-free survival (RFS) analysis of BMAL1 (**c**) and LDH-A (**d**) low and high breast cancer patients on the Kaplan–Meier plotter database. (p: log-rank, HR: hazard ratio). (**e**) Graphical summarization: tumor acidosis-mediated decrease of BMAL1 via inhibition of transcription activity and protein stability promotes metastatic potency, which could be prevented by melatonin that inhibits hypoxia-induced LDH-A in breast cancer.

We then investigated the possible clinical relevance of BMAL1 expression between normal and breast cancer tissues using the GSE database. BMAL1 was significantly decreased in breast cancer compared with normal breast tissue in GSE5364 and GSE3744 (Figure 6a). In the same GSE databases, LDH-A, which induces hypoxia-mediated acidosis, was also higher in cancer tissues (Figure 6b). We additionally investigated whether the BMAL1 gene was associated with survival in breast cancer patients using the Kaplan–Meier (KM) database [30]. When breast cancer was divided into BMAL1 and LDH-A low or high groups by the mean median value, recurrence free survival (RFS) was higher in the BMAL1 high group than the BMAL1 low group and lower in the LDH-A high group than the LDH-A low group (Figure 6c,d). Furthermore, RFS was higher in the CLOCK high group than the CLOCK low group. These databases predicted that breast cancer involves hypoxia-induced acidosis, which reduces BMAL1 and CLOCK. As a result, expression of BMAL1 and CLOCK was associated with poor prognoses in breast cancer patients. Overall, our results demonstrated that chronic hypoxia induced acidosis, one of the most obvious tumor microenvironments, which reduced the BMAL1 circadian clock gene via inhibition of transcriptional activity and decreased protein stability in breast cancer, and reduced BMAL1 promoted metastatic potency, which could be prevented by targeting tumor acidosis using melatonin via inhibition of LDH-A (Figure 6e). We additionally suggest a possibility that CLOCK is also reduced under hypoxia-mediated acidosis and reduced CLOCK promotes breast cancer metastasis.

## 4. Discussion

The majority of people in the world have abnormal circadian rhythms due to irregular living patterns. The disruption of circadian rhythms and a decrease of genes are highly associated with various diseases, including cancer. For example, recent studies have shown that night workers such as nurses are more likely to suffer from hormone-dependent cancers such as breast cancer [56,57]. Therefore, it can be expected that maintaining circadian patterns or genes is a strategy to prevent and treat cancer. Breast cancer is a prevalent female cancer and can sometimes be successfully treated with chemotherapy, radiation therapy, and surgery. However, when the tumor migrates and invades peripheral tissues, the survival rate is dramatically reduced [5]. There has been extensive research to overcome breast cancer metastasis, but it has not been adequately solved. According to previous reports, circadian genes, which are significantly reduced in cancer, suppress tumor progression including metastasis [16,17,18,19]. For this reason, we wanted to find a way to recover the reduced circadian genes in cancer to increase the survival rate by preventing metastasis.

The previous study reported that the expression patterns of the circadian genes were disrupted in tumor or adjacent-tumor tissue compared to normal tissue, and it was suggested that tumor macro or/and microenvironments are the cause [20]. Tumor hypoxia and acidosis are a characteristic of the tumor microenvironment in all solid tumors, and is clinically associated with tumor progression and poor prognoses in breast cancer patients [58,59]. In the previous study, acidification reduces circadian genes through the mTOR signaling pathway [29], but it is not well known yet in breast cancer, and it is not enough to explain the mTOR signaling pathway alone. In the present study, we made effort to find a new mechanism by which BMAL1 was reduced by the cancer microenvironment, and we additionally found that tumor hypoxia-induced acidosis significantly reduced the BMAL1 circadian clock gene via inhibition of both transcriptional activity and protein stability. Therefore, we suggested that circadian genes and rhythms were greatly influenced by pH.

HIFs are transcription factors that are activated in the hypoxic conditions. In the previous studies, HIF-1α reciprocally regulates circadian genes, both in vitro and in vivo models [29,31,60]. However, in breast cancer cells, both overexpression of HIF-1α and hypoxia-mimetic agent CoCl_2_ did not affect the BMAL1 protein expression. Therefore, we suggested that in breast cancer cells, a hypoxia-mediated decrease of BMAL1 protein expression is pH dependent and HIF-1 α independent.

Melatonin is mainly produced and secreted by the pineal gland, and plays a central role in the generation and regulation of circadian rhythm in humans [44]. In previous studies, melatonin has been shown to suppress cancer progression by inducing apoptosis and inhibiting angiogenesis, metastasis, and cell proliferation [50]. It also prevented disruption of the circadian rhythm in melanoma-bearing mice [61]. However, the relationship between reduced BMAL1 in acidosis and melatonin remains unclear. Interestingly, we found that hypoxia-induced acidic pH was buffered by melatonin through inhibition of LDH-A. We therefore suggest that melatonin is a way to recover the reduced circadian genes in cancer. We expect that other drugs and substances that maintain the acidified pH at the normal pH in cancer, or inhibit the tumor acidification process, can potentially recover circadian genes that are reduced under tumor acidosis.

In summary, we showed that tumor hypoxia-induced acidosis reduced the BMAL1 circadian clock gene in breast cancer. BMAL1 could be maintained in a tumor acidic pH by selectively targeting for acidosis via buffering the increased protons using NaHCO_3_ or inhibiting anaerobic glycolysis enzymes such as LDH-A using melatonin. These treatments provide a novel mechanism for inhibiting breast cancer metastasis by maintaining circadian gene BMAL1 in tumor hypoxia-induced acidosis.

## Supplementary Materials

The following are available online at https://www.mdpi.com/2073-4409/9/4/989/s1, Figure S1: Chronic hypoxia reduces the BMAL1/CLOCK expression independently of HIF-1α in breast cancer cells, Figure S2: Cell viability of NaOH and NaHCO3, Figure S3: BMAL1/CLOCK were reduced by tumor acidosis in breast cancer cells, Figure S4: Reduced BMAL1/CLOCK promotes metastasis in breast cancer cells, Figure S5: Melatonin prevents hypoxia-mediated decrease of BMAL1 by inhibiting LDH-A in breast cancer cells, Figure S6: RFS is higher in the CLOCK high group than the CLOCK low group in breast cancer patients, Figure S7: Densitometric measurements of Western blots in Figure 1, Figure S8: Densitometric measurements of Western blots in Figure 2, Figure S9: Densitometric measurements of Western blots in Figure 3, Figure S10: Densitometric measurements of Western blots in Figure 4, Figure S11: Densitometric measurements of Western blots in Figure 5, Table S1: Subtypes of human breast cancer cell lines, Table S2: Extracellular pH of normal and tumor, Table S3: Oligonucleotide sequences for the quantitative RT-PCR, Table S4: Media pH of indicated conditions in Figure 2, Table S5: Media pH of indicated conditions in Figure 3 and Supplementary Figure S3, Table S6: Media pH of indicated conditions in Figure 5.
