# Supplementary material for: Extracellular Acidosis Promotes Metastatic Potency via Decrease of the BMAL1 Circadian Clock Gene in Breast Cancer
Source: Cells. 2020 Apr 16;9(4):989. doi: 10.3390/cells9040989 (PMC7226966; doi:10.3390/cells9040989)
Supplement: Supplementary file 1 [file cells-09-00989-s001.pdf]

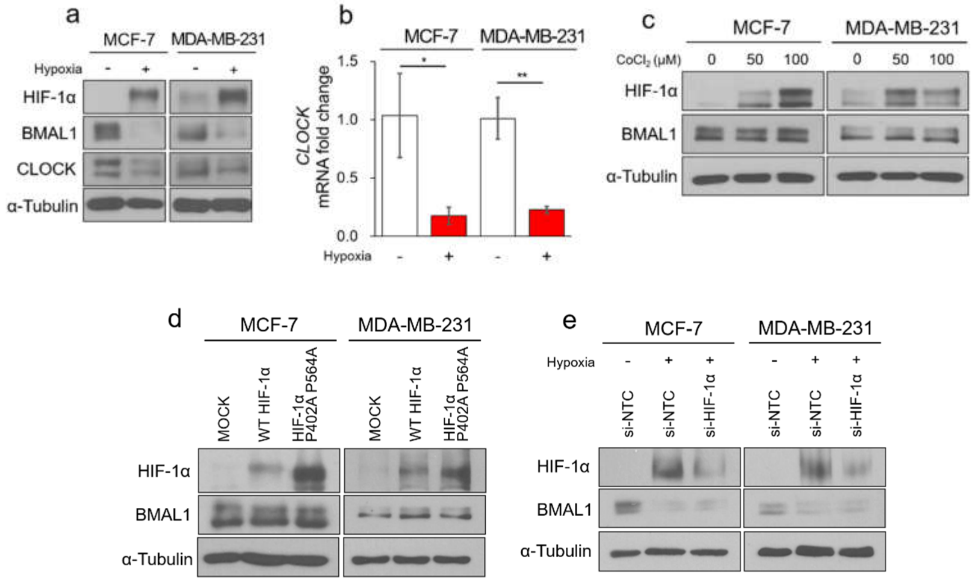


**Supplementary Figure 1.** Chronic hypoxia reduces the BMAL1/CLOCK expression independently of HIF-1$\alpha$ in breast cancer cells. (a, b) MCF-7 and MDA-MB-231 were incubated in normoxia or 2% $O_2$ hypoxia for 48 h. Cell lysates were analyzed by immunoblotting (a) and RT-qPCR (b). (c) MCF-7 and MDA-MB-231 were incubated in normoxia with $CoCl_2$ for 48 h. Cell lysates were analyzed by immunoblotting. (d) MCF-7 and MDA-MB-231 were transfected with HIF-1$\alpha^{WT}$ or HIF-1$\alpha^{P402A/P564A}$ plasmid, and incubated in normoxia for 48 h. Cell lysates were analyzed by immunoblotting. (e) MCF-7 and MDA-MB-231 were transfected with si-HIF-1$\alpha$, and incubated in 2% $O_2$ hypoxia for 48 h. Cell lysates were analyzed by immunoblotting.

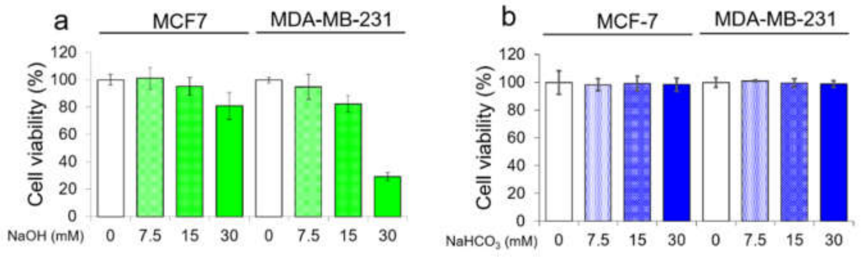


**Supplementary Figure 2.** Cell viability of NaOH and $NaHCO_3$. (a) MCF-7 and MDA-MB-231 were treated with NaOH for 24 h. Cell viability was measured by MTT assay. (b) MCF-7 and MDA-MB-231 were treated with $NaHCO_3$ for 24 h. Cell viability was measured by MTT assay.

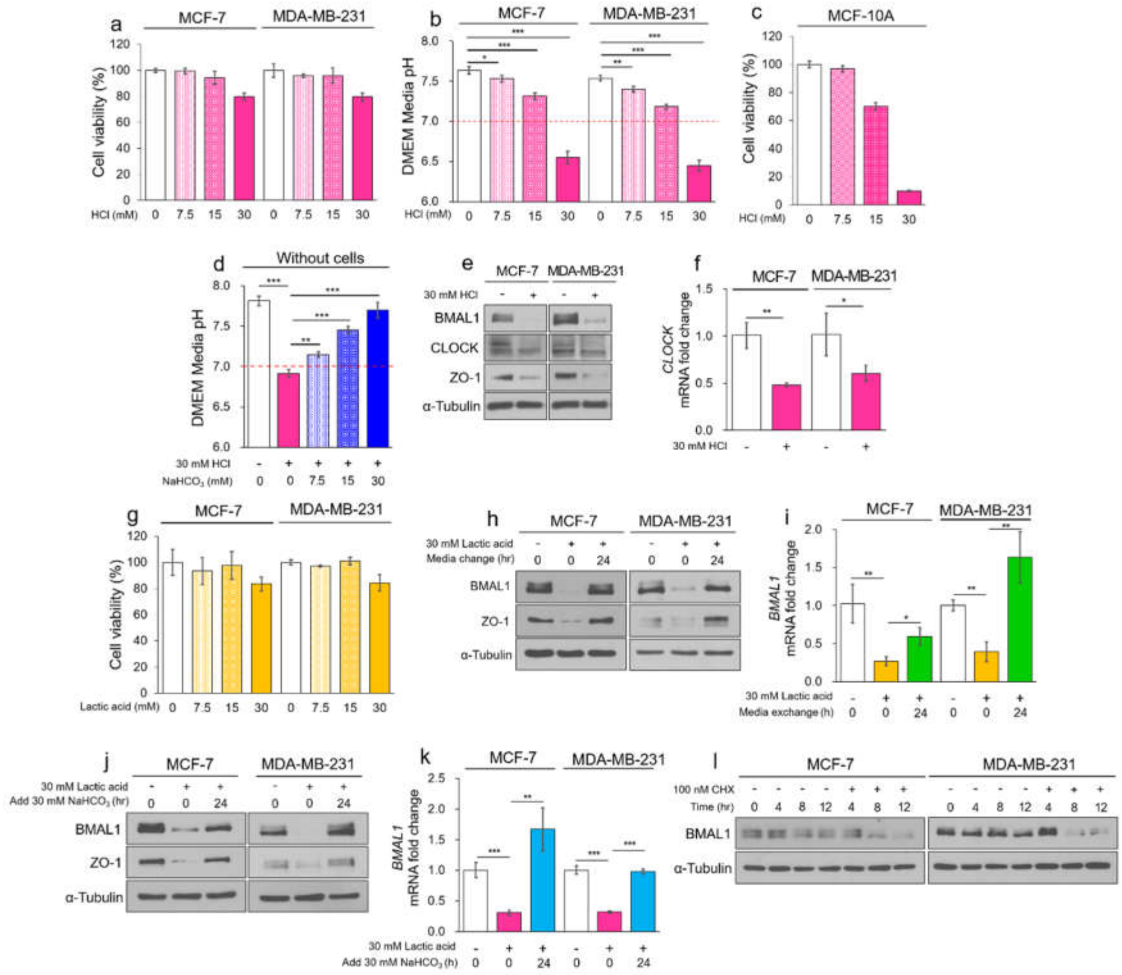

**Supplementary Figure 3.** BMAL1/CLOCK were reduced by tumor acidosis in breast cancer cells. (a) MCF-7 and MDA-MB-231 were treated with HCl-induced acidic media for 24 h. Cell viability was measured by MTT assay. (b) MCF-7 and MDA-MB-231 were treated with HCl-induced acidic media for 24 h. pH of the cultured media was immediately measured using pH meter. (c) MCF-10A were treated with HCl-induced acidic media for 24 h. Cell viability was measured by MTT assay. (d) The acidic cultured media were treated with $NaHCO_3$ and incubated for 24 h. pH of the cultured media was immediately measured using pH meter. (e, f) MCF-7 and MDA-MB-231 were treated with HCl-induced acidic media for 24 h. Cell lysates were analyzed by immunoblotting (e) and RT-qPCR (f). (g) MCF-7 and MDA-MB-231 were treated with lactic acid-induce acidic media for 48 h. Cell viability was measured by MTT assay. (h, i) MCF-7 and MDA-MB-231 were treated with lactic acid-induced acidic media for 48 h. The acidic cultured media were exchanged to fresh media and then incubated for 24 h. Cell lysates were analyzed by immunoblotting (h) and RT-qPCR (i). (j, k) MCF-7 and MDA-MB-231 were treated with lactic acid induced-acidic media for 48 h. The acidic cultured media were added to $NaHCO_3$ and then incubated for 24 h. Cell lysates were determined by immunoblotting (j) and RT-qPCR (k). (l) MCF-7 and MDA-MB-231 were treated with CHX for the indicated periods. Cell lysates were analyzed by immunoblotting. Data represent the mean ± SD, n = 3. *p < 0.05, **p < 0.01 and ***p < 0.001 vs. the control group or between two groups by Student's t-test.

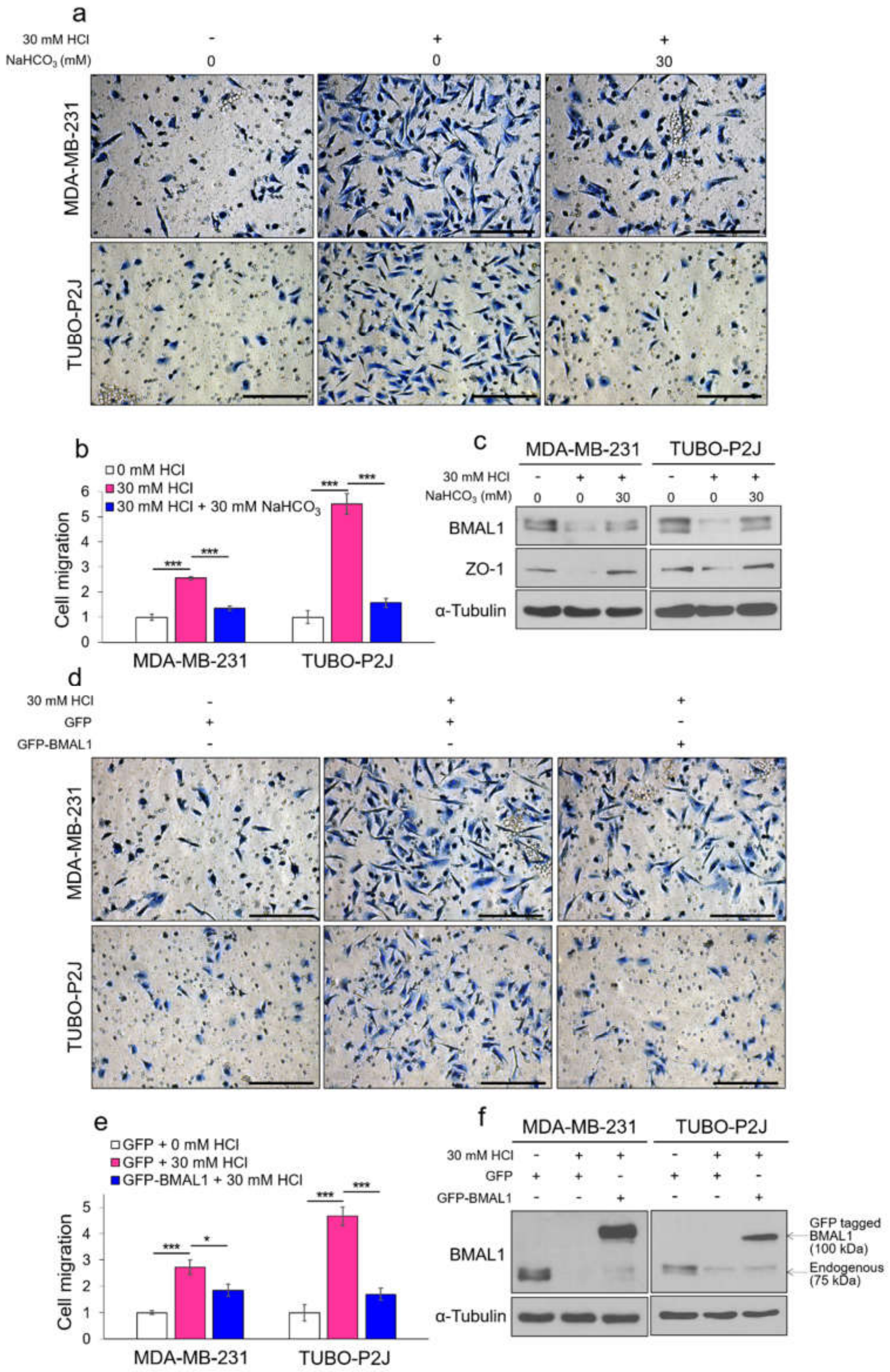


Supplementary figure 4 continued on next page

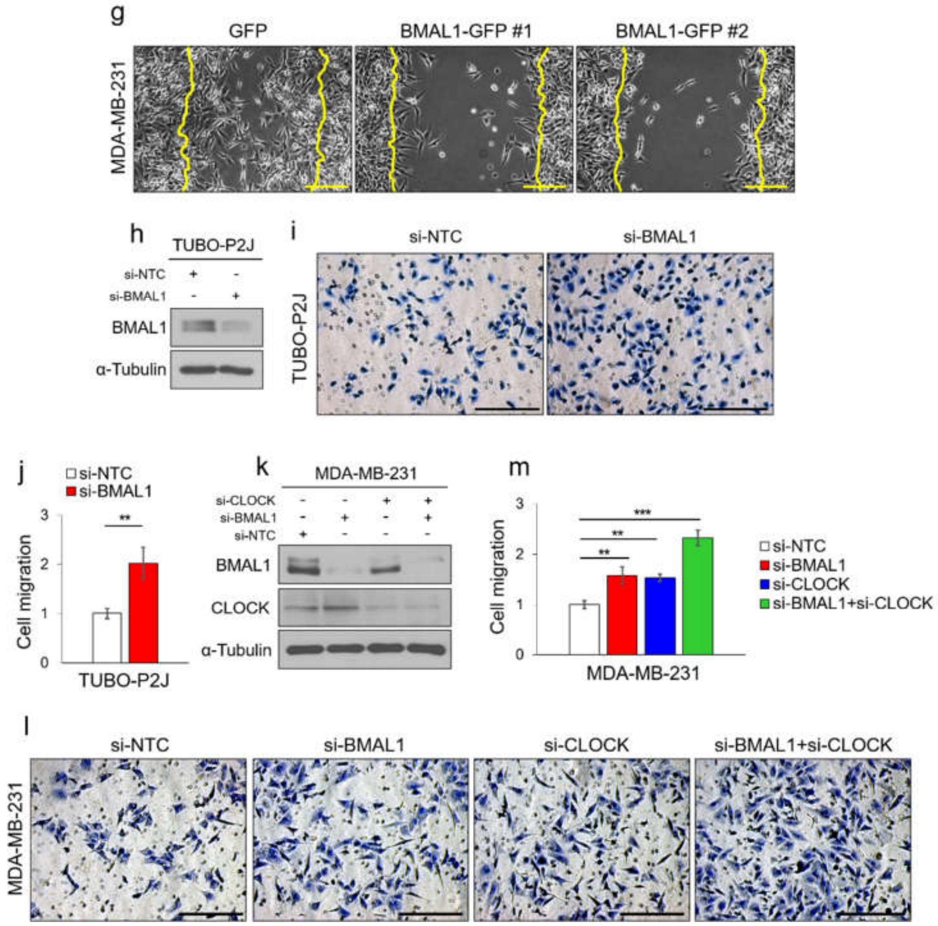


**Supplementary Figure 4.** Reduced BMAL1/CLOCK promotes metastasis in breast cancer cells. (a, b) MDA-MB-231 and TUBO-P2J were subjected to trans-well migration assay in HCl-mediated acidic condition with $NaHCO_3$ for 24 h as indicated. Representative images of migrated cells are shown. Scale bars:250μm (a). The average number of migrated MDA-MB-231 cells and TUBO-P2J cells was counted in three random microscopic fields (b). (c) MDA-MB-231 and TUBO-P2J were incubated in HCl-mediated acidic condition with $NaHCO_3$ for 24 h as indicated. Cell lysates were determined by immunoblotting. (d, e) MDA-MB-231 and TUBO-P2J were transfected with GFP or GFP-BMAL1, and subjected to trans-well migration assay in acidic condition for 24 h as indicated. Representative images of migrated cells are shown. Scale bars:250μm (d) and the average number of migrated MDA-MB-231 and TUBO-P2J cells was counted in three random microscopic fields (e). (f) MDA-MB-231 and TUBO-P2J were transfected with GFP or GFP-BMAL1, and incubated in acidic condition for 24 h as indicated. Cell lysates were analyzed by immunoblotting. (g) GFP or GFP-BMAL1 stably overexpressed MDA-MB-231 cell lines were subjected to wound-healing assay for 48 h. Representative images of migrated cells are shown. Scale bars:250μm. (h) TUBO-P2J was transfected with si-NTC or si-BMAL1. Cell lysates were analyzed by immunoblotting. (i, j) TUBO-P2J was transfected with si-NTC or si-BMAL1, and subjected to trans-well migration assay for 24 h. Representative images of migrated cells are shown. Scale bars:250μm (i) and the average number of migrated TUBO-P2J cells was counted in three random microscopic fields (j). (k) MDA-MB-231 was transfected with si-BMAL1 and/or si-CLOCK. Cell lysates were analyzed by immunoblotting. (l, m) MDA-MB-231 was transfected with si-NTC, si-BMAL1 and/or si-CLOCK, and subjected to trans-well migration assay for 24 h. Representative images of migrated cells are shown. Scale bars:250μm (l) and the average number of migrated MDA-MB-231 cells was counted in three random microscopic fields (m). Data represent the mean ± SD, n = 3. $^*p < 0.05$, $^{**}p < 0.01$ and $^{***}p < 0.001$ vs. the control group or between two groups by Student's t-test.

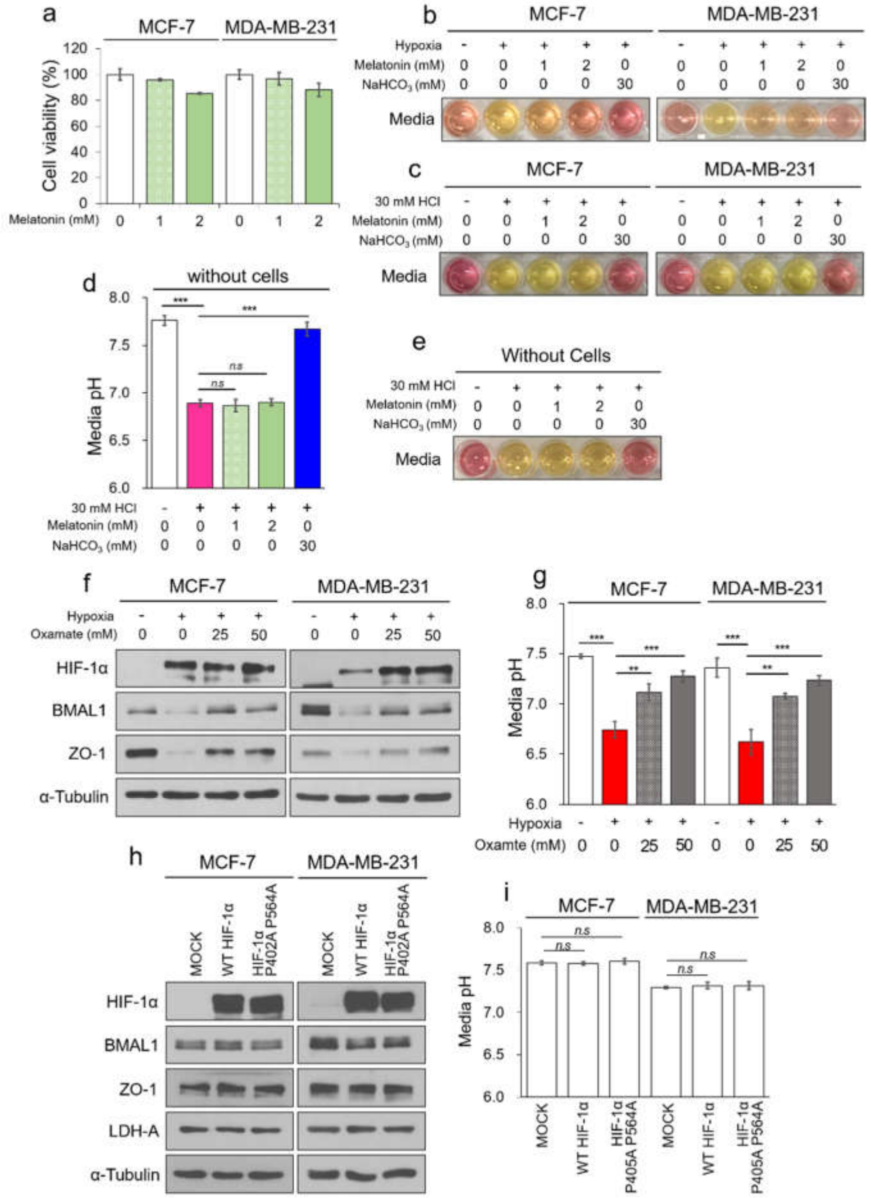


**Supplementary Figure 5.** Melatonin prevents hypoxia-mediated decrease of BMAL1 by inhibiting LDH-A in breast cancer cells. (a) MCF-7 and MDA-MB-231 were treated with melatonin for 48 h. Cell viability was measured by MTT assay. (b, c) MCF7 and MDA-MB-231 were incubated in normoxia or 2% $O_2$ hypoxia for 48 h (b) or HCl-induced acidic condition for 24 h (c) with melatonin or $NaHCO_3$. Representative images of the cultured media are shown on the indicated conditions. (d, e) The HCl-induced acidic media were treated with melatonin or $NaHCO_3$ for 24 h. pH of the media was immediately measured using pH meter (d) and representative images of pH of the media are shown (e). (f, g) MCF-7 and MDA-MB-231 were incubated in normoxia or 2% $O_2$ hypoxia with oxamate for 48 h. Cell lysates were analyzed by immunoblotting (f) and pH of the cultured media was immediately measured using pH meter (g). (h, i) MCF-7 and MDA-MB-231 were transfected with HIF-1$\alpha^{WT}$ or HIF-1$\alpha^{P402A/P564A}$ plasmid, and incubated in normoxia for 48 h. Cell lysates were analyzed by immunoblotting (h) and pH of the cultured media was immediately measured using pH meter (i). Data represent the mean ± SD, n = 3. **$p < 0.01$ and ***$p < 0.001$ vs. the control group or between two groups by Student's t-test.

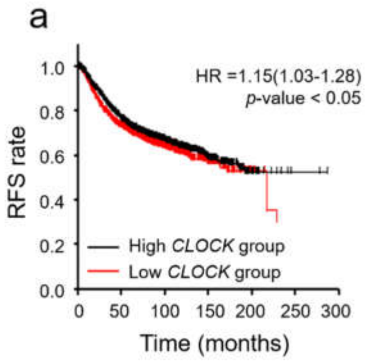


**Supplementary Figure 6.** RFS is higher in the CLOCK high group than the CLOCK low group in breast cancer patients. (a) Relapse-free survival (RFS) analysis of CLOCK low and high breast cancer patients on Kaplan-Meier plotter database. (p:log-rank, HR:hazard ratio).

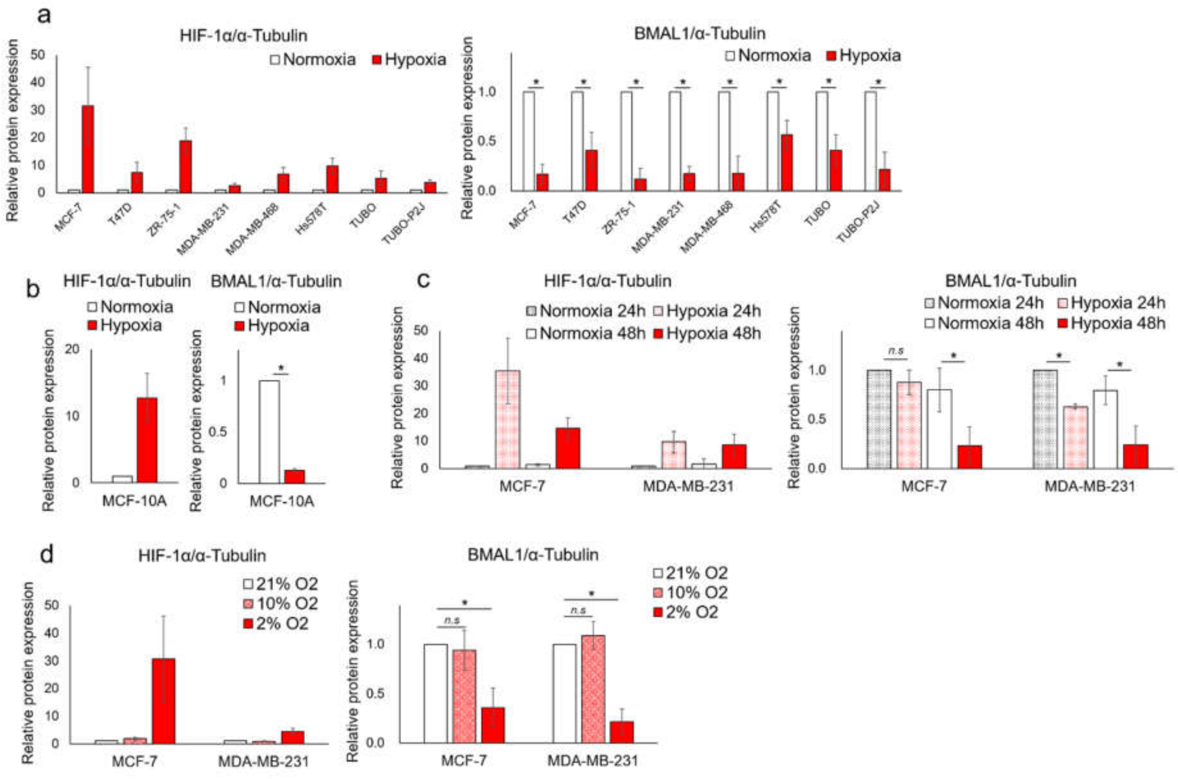


**Supplementary Figure 7**. Densitometric measurements of western blots in figure 1. (a) Densitometry of western blot bands in figure 1a. (b) Densitometry of western blot bands in figure 1b. (c) Densitometry of western blot bands in figure 1c. (d) Densitometry of western blot bands in figure 1d. Data represent the mean ± SD, n = 3. *p < 0.05 vs. the control group or between two groups by Student's t-test.

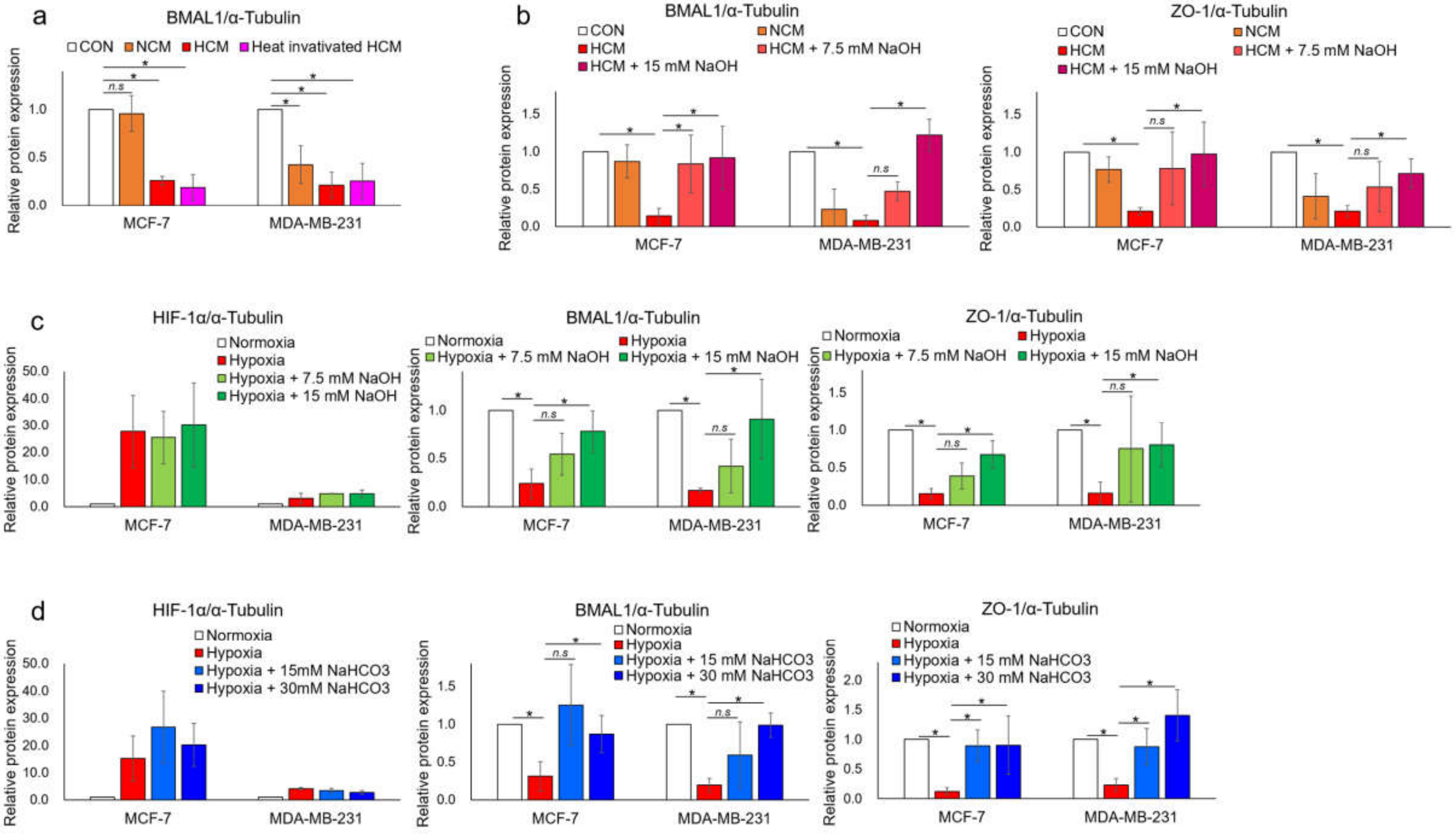


**Supplementary Figure 8**. Densitometric measurements of western blots in figure 2. (a) Densitometry of western blot bands in figure 2a. (b) Densitometry of western blot bands in figure 2b. (c) Densitometry of western blot bands in figure 2c. (d) Densitometry of western blot bands in figure 2e. Data represent the mean ± SD, n = 3. *$p < 0.05$ vs. the control group or between two groups by Student's t-test.

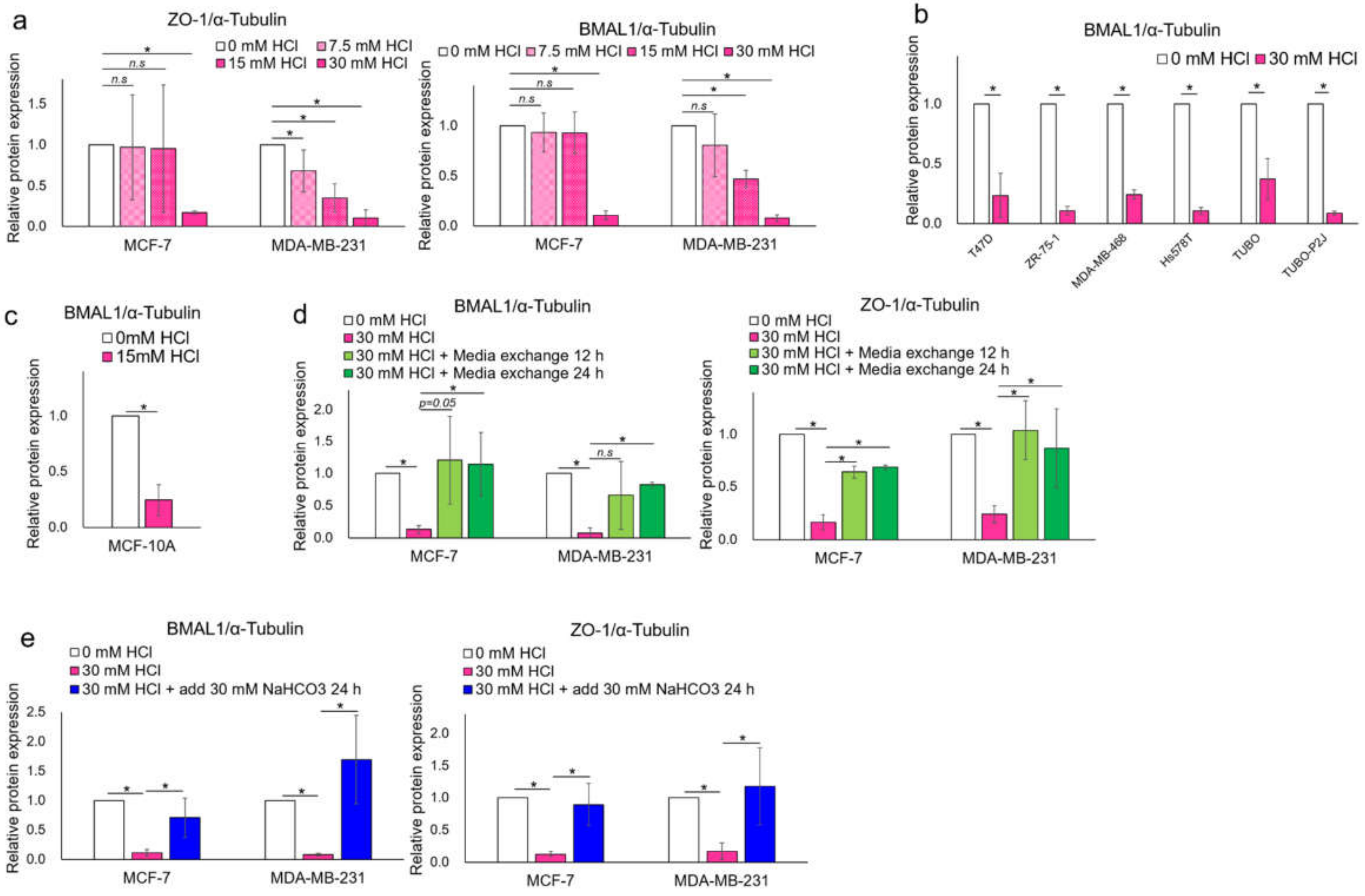


**Supplementary Figure 9**. Densitometric measurements of western blots in figure 3. (a) Densitometry of western blot bands in figure 3c. (b) Densitometry of western blot bands in figure 3d. (c) Densitometry of western blot bands in figure 3f. (d) Densitometry of western blot bands in figure 3g. (e) Densitometry of western blot bands in figure 3i. Data represent the mean ± SD, n = 3. *$p < 0.05$ vs. the control group or between two groups by Student's t-test.

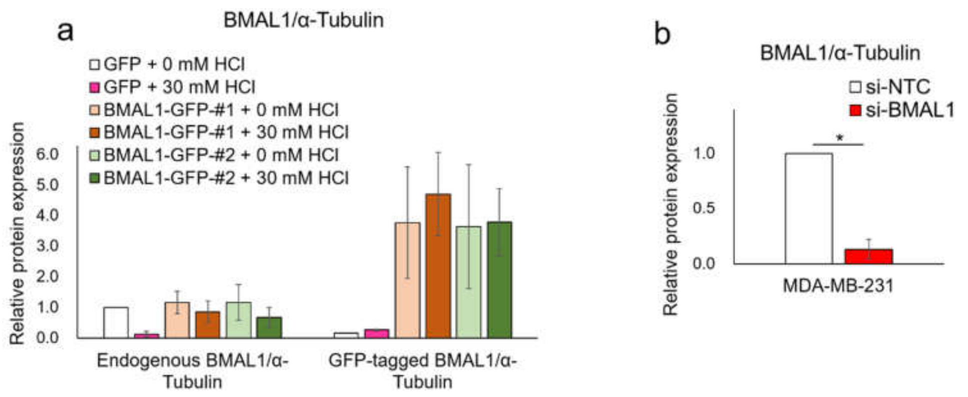


**Supplementary Figure 10**. Densitometric measurements of western blots in figure 4. (a) Densitometry of western blot bands in figure 4a. (b) Densitometry of western blot bands in figure 4d. Data represent the mean ± SD, n = 3. *p < 0.05 vs. the control group or between two groups by Student's t-test.

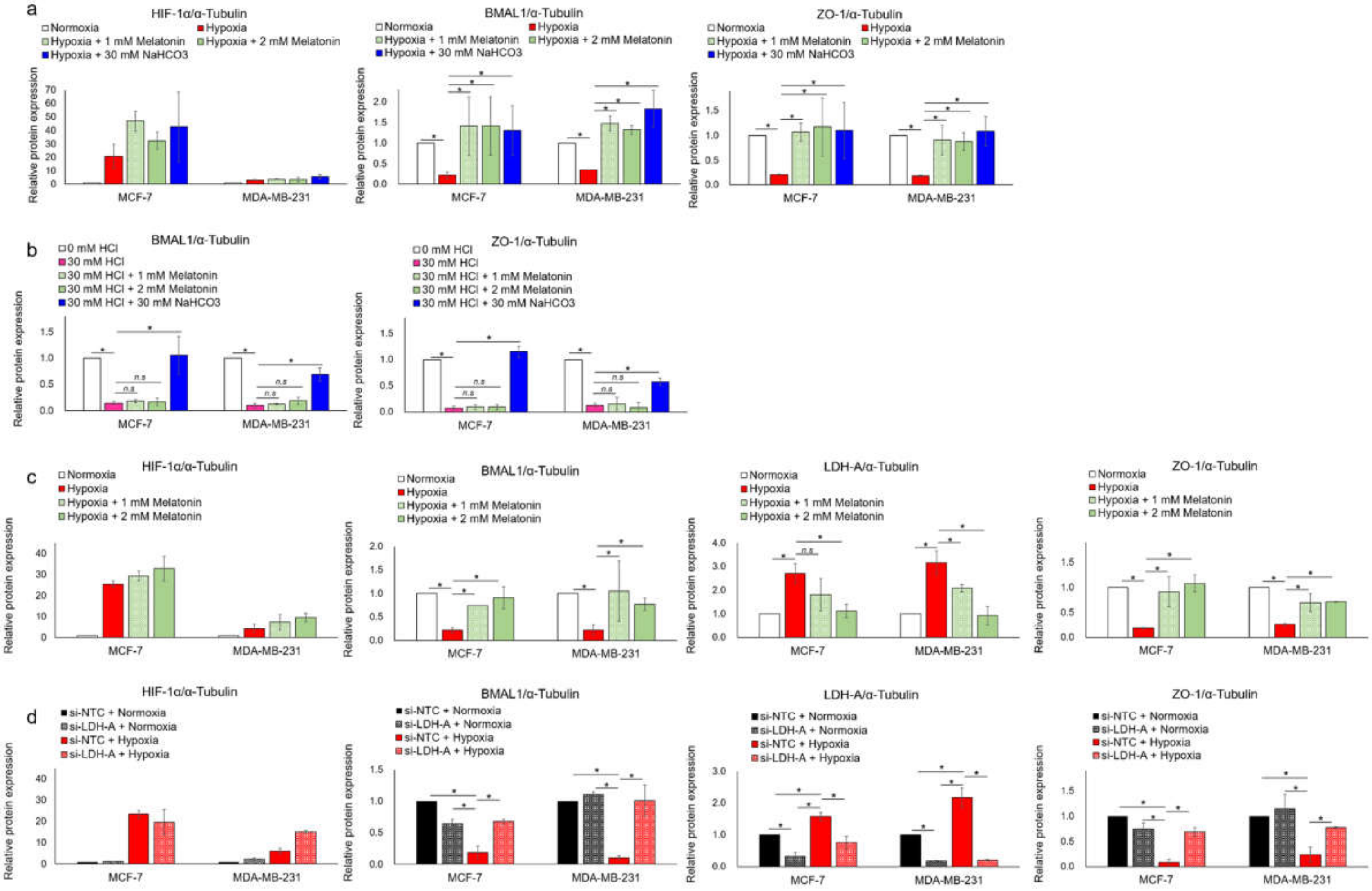


**Supplementary Figure 11**. Densitometric measurements of western blots in figure 5. (a) Densitometry of western blot bands in figure 5a. (b) Densitometry of western blot bands in figure 5c. (c) Densitometry of western blot bands in figure 5e. (d) Densitometry of western blot bands in figure 5f. Data represent the mean ± SD, n = 3. *p < 0.05 vs. the control group or between two groups by Student's t-test.

**Supplementary Table 1.** Subtypes of human breast cancer cell lines.

| Classification | Immunoprofile | Example cell lines |
|---|---|---|
| Luminal A | $ER^{+}$, $PR^{+/-}$, $HER2^{-}$ | MCF-7, T47D |
| Luminal B | $ER^{+}$, $PR^{+/-}$, $HER2^{+}$ | ZR-75-1 |
| Basal-like | $ER^{-}$, $PR^{-}$, $HER2^{-}$ | MDA-MB-468, MDA-MB-231, Hs578T |

**Supplementary Table 2.** Extracellular pH of normal and tumor.

| Normal | Tumor | ref. |
|---|---|---|
| 7.5 | 6.5-6.8 | *Biomater Res,* **22,** 1-11 (2018) [34] |
| 7.4 | 6.8 | *Cell Rep,* **18,** 2228-2242 [35] |
| 7.4 | 6.2-7.0 | *Clin Cancer Res,* **21,** 4502-4504 (2015) [36] |
| 7.4 | 6.7 | *Front Physiol,* **4,** 370 (2013) [37] |
| 7.2-7.4 | 6.5-6.9 | *Cancer Res,* **73,** 1524-1535 (2013) [38] |
| 7.4 | 6.8-7.1 | *Nat Rev Cancer,* **11,** 671 (2011) [39] |

**Supplementary Table 3.** Oligonucleotide sequences for the quantitative RT-PCR.

| Name | | Sequence ( 5' -> 3' ) |
|---|---|---|
| BMAL1 (human) | Forward | TGCCACCAATCCATACACAG |
| | Reverse | TCGGTCACATCCTACGACAAAC |
| CLOCK (human) | Forward | CTCATCTGCTGGAAAGTGATTC |
| | Reverse | TGGCTCCTTTGGGTCTATTG |
| β-actin (human) | Forward | AAATCTGGCACCACACCTTC |
| | Reverse | GGGGTGTTGAAGGTCTCAAA |
| α-Tubulin (human) | Forward | CTTCGTCTCCGCCATCAG |
| | Reverse | CGTGTTCCAGGCAGTAGAGC |

**Supplementary Table 4.** Media pH of indicated conditions in figure 2

| | | | | | | |
|---|---|---|---|---|---|---|
| CON | + | - | - | - | - | Figure 2b |
| NCM | - | + | - | - | - | |
| HCM | - | - | + | + | + | |
| NaOH (mM) | 0 | 0 | 0 | 7.5 | 15 | |
| MCF-7 | 7.70(±0.09) | 7.39(±0.07) | 6.74(±0.07) | 7.21(±0.08) | 7.43(±0.07) | |
| MDA-MB-231 | 7.43(±0.07) | 6.90(±0.04) | 6.47(±0.07) | 6.95(±0.13) | 7.29(±0.05) | |

| | | | | | |
|---|---|---|---|---|---|
| Hypoxia | - | + | + | + | Figure 2c |
| NaOH (mM) | 0 | 0 | 7.5 | 15 | |
| MCF-7 | 7.59(±0.12) | 6.83(±0.10) | 7.28(±0.11) | 7.55(±0.02) | |
| MDA-MB-231 | 7.37(±0.09) | 6.54(±0.16) | 6.86(±0.10) | 7.18(±0.10) | |

| | | | | | |
|---|---|---|---|---|---|
| Hypoxia | - | + | + | + | Figure 2e |
| $NaHCO_3$ (mM) | 0 | 0 | 15 | 30 | |
| MCF-7 | 7.66(±0.11) | 6.85(±0.10) | 7.36(±0.07) | 7.72(±0.05) | |
| MDA-MB-231 | 7.32(±0.11) | 6.52(±0.17) | 7.20(±0.07) | 7.60(±0.07) | |

**Supplementary Table 5.** Media pH of indicated conditions in figure 3 and supplementary figure 3

| HCl (mM) | 0 | 7.5 | 15 | 30 | |
|---|---|---|---|---|---|
| MCF-7 | 7.64(±0.05) | 7.53(±0.04) | 7.32(±0.04) | 6.55(±0.06) | Figure 3c |
| MDA-MB-231 | 7.54(±0.04) | 7.40(±0.04) | 7.18(±0.03) | 6.45(±0.07) | |

| 30 mM HCl | - | + | + | + | |
|---|---|---|---|---|---|
| Media change (hr) | 0 | 0 | 12 | 24 | |
| MCF-7 | 7.70(±0.07) | 6.69(±0.14) | 7.86(±0.07) | 7.73(±0.05) | Figure 3g |
| MDA-MB-231 | 7.52(±0.09) | 6.36(±0.12) | 7.20(±0.07) | 7.60(±0.07) | |

| 30 mM HCl | 0 | 0 | 24 | |
|---|---|---|---|---|
| Add 30 mM $NaHCO_3$ (hr) | - | + | + | |
| MCF-7 | 7.67(±0.03) | 6.73(±0.13) | 7.60(±0.09) | Figure 3i |
| MDA-MB-231 | 7.32(±0.11) | 6.52(±0.17) | 7.44(±0.07) | |

| 30 mM Lactic acid | - | + | + | |
|---|---|---|---|---|
| Media change (hr) | 0 | 0 | 24 | |
| MCF-7 | 7.45(±0.07) | 6.80(±0.03) | 7.61(±0.03) | Supplementary Figure 3h |
| MDA-MB-231 | 7.26(±0.05) | 6.48(±0.08) | 7.54(±0.04) | |

| 30m M Lactic acid | - | + | + | |
|---|---|---|---|---|
| Add 30 mM $NaHCO_3$ (hr) | 0 | 0 | 24 | |
| MCF-7 | 7.52(±0.03) | 6.75(±0.06) | 7.58(±0.06) | Supplementary Figure 3j |
| MDA-MB-231 | 7.26(±0.06) | 6.38(±0.07) | 7.44(±0.04) | |

**Supplementary Table 6.** Media pH of indicated conditions in figure 5

| | | | | | | |
|---|---|---|---|---|---|---|
| Hypoxia | - | + | + | + | + | Figure 5a |
| Melatonin (mM) | 0 | 0 | 1 | 2 | 0 | |
| $NaHCO_3$ (mM) | 0 | 0 | 0 | 0 | 30 | |
| MCF-7 | 7.47(±0.03) | 6.71(±0.15) | 7.06(±0.04) | 7.23(±0.07) | 7.47(±0.07) | |
| MDA-MB-231 | 7.35(±0.09) | 6.53(±0.13) | 7.01(±0.10) | 7.27(±0.03) | 7.46(±0.08) | |
| 30 mM HCl | - | + | + | + | + | Figure 5c |
| Melatonin (mM) | 0 | 0 | 1 | 2 | 0 | |
| $NaHCO_3$ (mM) | 0 | 0 | 0 | 0 | 30 | |
| MCF-7 | 7.66(±0.03) | 6.65(±0.07) | 6.64(±0.09) | 6.62(±0.06) | 7.63(±0.07) | |
| MDA-MB-231 | 7.57(±0.05) | 6.49(±0.06) | 6.42(±0.02) | 6.42(±0.05) | 7.57(±0.07) | |
